# Editorial: Infection-Related Immune-Mediated Diseases and Microbiota

**DOI:** 10.3389/fped.2020.00108

**Published:** 2020-04-07

**Authors:** Kyung-Yil Lee, Hiromichi Hamada, Miika Arvonen

**Affiliations:** ^1^The Catholic University of Korea, College of Medicine, Seoul, South Korea; ^2^Department of Pediatrics, Tokyo Women's Medical University Yachiyo Medical Center, Chiba, Japan; ^3^Department of Pediatrics, Kuopio University Hospital, Kuopio, Finland

**Keywords:** microbiota, Kawasaki disease, juvenile idiopathic arthritis, Hirschsprung's disease, etiology, epidemiology, pathogenesis, multiple-drug resistant Staphylococcus aureus

Microbiota may form a collaborative partnership with the host. Eubiosis, i.e., the homeostatic state of the host and symbiotic microflora, is critical for maintaining a state of well-being in the host. Commensal bacteria may prevent by colonization by external pathogens, and the mucosal immune system, such as gut-associated lymphoid tissues (GALT), is established after colonization of the gut by normal flora, suggesting that microbiota and host immune systems are closely related to each other (1). On the other hand, certain strains of microbes can elicit infectious events on occasion when they invade into the host and induce an immune reaction.

The microbiota in an individual continuously changes after birth (2), and certain strains of microbes in ethnic groups may be influenced by environmental factors such as diet and socio-economic state. Thus, the microbiota is different in different populations and can be changed by the changing environment. The disruption of the reciprocal equilibrium between microbiota and host, that is, dysbiosis, has now become a major subject of study in various medical fields, including those of metabolic, gastroenteric, psychiatric, neurologic, and allergic diseases and cancers. Also, intestinal dysbiosis has been reported in patients with Kawasaki disease (KD) and juvenile idiopathic arthritis (JIA) (3, 4). Although the mechanisms relating dysbiosis and provocation of diseases remain elusive, over-production of toxic materials from dysbiosis-causing microbials, vulnerable invasion of microbials through wakened mucosal barriers, or the disruption of the homeostatic relationship between the microbiota and the host's immune system may be responsible for disease onset [(5), Esposito et al.].

The human gut microbiota is composed of over 1,000 species, and different strains are colonized in the small intestine and colon. Hirschsprung's disease (HD) is characterized by a defect of intestinal nerve ganglia, and occasionally patients with HD can be affected with HD-associated enterocolitis before or after operation. In the majority of cases, the causative agents of HD-associated enterocolitis are not external pathogens, and dysbiosis may be associated with intestinal inflammation (6). A different microbiome between patients with total colon resection and those with partial resection is observed, and the former group tends to have a higher risk of enterocolitis (Pini Prato et al.). Staphylococcus species are one of the main normal flora that colonize the skin and, on occasion, the vagina tract of pregnant women. Normal flora in neonates begin to colonize just after birth, though some strains may be different according to delivery method (Caesarian section or vaginal delivery). Although colonization by commensals, including multiple-drug-resistant *S. aureus*, may be a risk factor for subsequent infection, severe invasive infection is rare in immune-competent hosts. It is observed that colonized multiple-drug-resistant *S. aureus* in neonates could be transmitted from their mothers (Lin and Yao).

Childhood immune-mediated diseases, including KD and JIA, may be associated with prevalent infections in childhood. Interestingly, the incidence of both diseases is quite different among populations; the incidence of KD is over 10–20 times higher in East Asian countries such as Japan and Korea, and the incidence of JIA is over 10 times higher in North European countries compared to children in East Asian countries (7). The discrepancy of incidence rates across the populations has been observed in other infection-related immune-mediated diseases, including type I diabetes, inflammatory bowel disease, and Behcet disease. Although genetic or environmental factors may be responsible for the finding, it is possible that children living in higher-prevalence countries may have more chances of being exposed to KD or JIA pathogens, since the clinical manifestations and immune function of children with KD or JIA are nearly identical across the populations.

Based on the epidemiological and clinical characteristics of KD, it has been suggested that it may be associated with infectious agents, including viruses, especially RNA viruses (Rowley and Schulman), bacteria that can activate innate and adaptive immune systems (Nagata; Nakamura et al.), and strains of normal flora (Esposito et al.; Rhim et al.). Since KD is a self-limiting systemic inflammation, many laboratory parameters are affected during the natural course of the disease; levels of white blood cells, erythrocyte sedimentation rate, C-reactive protein, albumin, hemoglobin, and other biomarkers such as proinflammatory cytokines are up-regulated or down-regulated, and various genetic traits are related to KD susceptibility or phenotype (Chaudhary et al.). Intravenous immunoglobulin (IVIG) treatment is known to reduce the risk of coronary artery lesions (CALs), a major complication of KD. It was reported that a patient group with spontaneous defervescence had a higher rate of CALs (aneurysms) at 1 month after disease onset compared to the IVIG-treated group (Hu et al.). However, a small proportion of initially CAL-negative patients in both groups showed CALs at 1 month, suggesting that some KD patients may have ongoing inflammation in CALs after defervescence (8).

There are few human diseases of which the pathogenesis has been clearly proven from the era of Hippocrates to the present time. Although etiologic agents have been discovered in infectious diseases, the substances inducing inflammation and tissue cell injury in infectious diseases and infection-related immune-mediated diseases are not pathogens themselves but smaller substances derived from the infectious insults (9). Many researchers may agree on the notions that every disease has etiologic substances and that present immunological concepts have limitations in explaining the pathogenesis of many diseases. It is now known that the host immune system reacts not to only the substances derived from the infectious agents, including toxins and pathogen-associated molecular patterns (PAMPs), but also to the substances derived from host cells injured by infectious insults such as damage (danger)-associated molecular patterns (DAMPs), especially in cases of intracellular infection such as viral or intracellular pathogen infections (10). Because of the appearance of KD as a novel disease in East Asia, the discovery of the etiology and pathogenesis of KD will help to extend our understanding regarding these issues of human diseases. The pathogenesis of KD is associated with the immune reaction of the host against infectious insults. The contributions to this Research Topic present and discuss various aspects of the pathogenesis of KD, including the roles of components of the adaptive immune system such as IgA plasma cells and cytotoxic CD8 T cells against viral antigens (Rowley and Schulman), a similar/common immune process associated with the activation of T cells and innate immune cells caused by diverse pathogens (Nagata), an innate immune response including PAMPs, toll-like receptors, and complement pathways (Nakamura et al.), and types of DAMPs produced after infectious insults based on the protein-homeostasis-system hypothesis (Rhim et al.).

## Author Contributions

K-YL wrote the manuscript. HH and MA have made a substantial, direct, and intellectual contribution to the work. All authors approved it for publication.

### Conflict of Interest

The authors declare that the research was conducted in the absence of any commercial or financial relationships that could be construed as a potential conflict of interest.
